# In Silico Design and Evaluation of Quinone Methide Oxime Derivatives as Potential Non-Covalent Steroid Sulfatase Inhibitors

**DOI:** 10.3390/molecules31152612

**Published:** 2026-07-27

**Authors:** Dmytro Khylyuk, Oleg M. Demchuk, Sergii Holota, Dagmara Otto-Ślusarczyk, Marta Struga, Franciszek Burdan, Monika Wujec

**Affiliations:** 1 Chair and Department of Organic Chemistry, Medical University of Lublin, ul. Chodźki 4a, 20-093 Lublin, Poland; 2 Faculty of Medicine, The John Paul II Catholic University of Lublin, Konstantynow 1J, 20-708 Lublin, Poland; 3 Department of Pharmaceutical, Organic and Bioorganic Chemistry, Danylo Halytsky Lviv National Medical University, 69 Pekarska St., 79010 Lviv, Ukraine; golota_serg@yahoo.com; 4 Molecular Design Center, Danylo Halytsky Lviv National Medical University, Pekarska 69, 79010 Lviv, Ukraine; 5 Department of Biochemistry, Medical University of Warsaw, Banacha 1, 02-097 Warsaw, Poland; dagmara.otto@wum.edu.pl (D.O.-Ś.);; 6 Department of Human Anatomy, Medical University of Lublin, 4 Jaczewski Street, 20-090 Lublin, Poland

**Keywords:** steroid sulfatase, in silico drug design, breast cancer, sulfatase inhibitors, quinone methide oximes

## Abstract

Steroid sulfatase (STS) plays a crucial role in intratumoral estrogen biosynthesis and represents an attractive therapeutic target in estrogen receptor-positive breast cancer. In this study, a new series of potential STS inhibitors based on the quinone methide oxime scaffold, precisely 2-(4-hydroxyiminocyclohexa-2,5-dien-1-ylidene)-2-phenylacetonitrile framework, were designed and evaluated using an integrated in silico approach. A virtual library comprising 216 compounds (including syn/anti isomers) was screened by molecular docking against the human STS crystal structure (PDB ID: 8EG3). The binding affinities ranged from −7.077 to −9.726 kcal·mol^−1^; however, only the best-performing compound 45-syn showed values comparable to those of the reference ligands. The top-ranked compound (45-syn) exhibited favorable interactions within the catalytic site, including polar contacts near the FGly–Ca^2+^ region and extensive hydrophobic and π–π interactions in the adjacent pocket. Structure–binding relationship analysis highlighted the importance of electron-withdrawing substituents at R^1^ and aromatic moieties at R^2^ for enhanced binding. Molecular dynamics simulations confirmed the stability of ligand–STS complexes and demonstrated reduced flexibility compared to the apo form. Additionally, in silico ADMET predictions indicated generally favorable drug-like profiles for selected candidates. Overall, the results highlight computationally prioritized scaffolds that merit further synthesis and biological evaluation as potential STS inhibitors.

## 1. Introduction

Despite considerable advances in oncology and medicinal chemistry, improving the effectiveness and durability of cancer treatment remains a central challenge [1]. Breast cancer, the most frequently diagnosed malignancy in women worldwide and a leading cause of cancer mortality, is driven in a substantial proportion of cases by estrogen receptor (ER) signaling. Approximately 70% of breast tumors are ER positive (ER+), reflecting a dependency on intricate genomic and rapid non-genomic ERα-mediated networks [2]. At the genomic level, ligand-bound ERα orchestrates transcriptional programs governing proliferation, survival, and cell cycle progression; extranuclear ERα signaling concurrently activates PI3K/AKT and MAPK pathways through membrane proximal complexes and crosstalk with receptor tyrosine kinases such as HER2 and IGF1R [3,4,5]. These multilayered circuits foster tumor growth and adaptability and are further complicated by recurrent ESR1 mutations (notably Y537S and D538G), which confer ligand-independent receptor activity and contribute to endocrine resistance [6,7]. Collectively, these features underscore the clinical imperative to disrupt both ER signaling and the steroidogenic sources that sustain it [8].

Steroid sulfatase (STS) has consequently emerged as an attractive therapeutic target in hormone-dependent malignancies [9,10,11]. STS regenerates bioactive steroids from their sulfated precursors by hydrolyzing estrone sulfate (E1S) and dehydroepiandrosterone sulfate (DHEA-S) to estrone (E1), estradiol (E2), and androstenediol—metabolites that can drive proliferation in estrogen-responsive tissues. In postmenopausal breast cancer, where circulating E1S constitutes the dominant estrogen reservoir, the sulfatase pathway becomes a principal source of intratumoral estrogens [12,13]. Consistent with this biology, elevated STS expression and activity are frequently observed in breast and endometrial tumors and associate with adverse outcomes, highlighting both mechanistic and translational relevance [14].

Progress in STS-directed medicinal chemistry has been catalyzed by structural elucidation of the enzyme. STS is an integral endoplasmic reticulum membrane protein whose active site contains a formylglycine (FGly) gem diol coordinated to Ca^2+^ [15,16]. Crystallographic and biochemical studies show that aryl sulfamates engage this pocket and cause time-dependent, covalent inactivation via transfer of the sulfamate group to the catalytic machinery. These data establish why an unsubstituted aryl sulfamate N–H is required for irreversible inactivation and why bulky hydrophobic substituents—often positioned to mimic steroid CD rings—enhance binding and potency by engaging hydrophobic regions adjacent to the catalytic cavity [17,18].

In sum, STS operates as a gatekeeper of intratumoral steroidogenesis and a critical node in ER-dependent tumor biology. Convergent biochemical, structural, and pharmacological evidence supports selective STS inhibition as a rational component of modern endocrine therapy aimed at deep estrogen deprivation and durable suppression of ER-driven signaling in estrogen-dependent malignancies [19,20].

Non-covalent (reversible) sulfatase inhibitors interact with the enzyme active site through weak intermolecular forces and can dissociate dynamically, unlike covalent inhibitors that irreversibly inactivate the enzyme [21]. A major advantage of non-covalent inhibitors is their improved safety profile, as they are less likely to form off-target covalent adducts and therefore exhibit reduced toxicity risks. Their reversible binding allows fine-tuning of enzyme inhibition and avoids prolonged suppression of physiological processes such as hormone regulation.

Additionally, non-covalent inhibitors provide greater flexibility in medicinal chemistry optimization and enable higher selectivity through specific intermolecular interactions.

Thus, for therapeutic targeting of sulfatases, especially in chronic diseases, non-covalent inhibitors may represent a safer and more controllable alternative to irreversible covalent compounds, although the safety profile of a drug is determined by multiple factors [22,23].

Given the central importance of STS in sustaining ER-driven tumor growth, the search for potent and selective inhibitors increasingly relies on advances in modern medicinal chemistry. Integrating computational modeling with rational ligand design allows rapid exploration of chemical space and early prediction of inhibitory potential. Such in silico approaches streamline the identification of promising anticancer candidates before committing to synthesis and biological evaluation.

With the aim of developing a new class of non-covalent steroid sulfatase inhibitors, we designed and evaluated an in silico library of novel compounds derived from the quinone methide oxime scaffold, precisely 2-(4-hydroxyiminocyclohexa-2,5-dien-1-ylidene)-2-phenyl acetonitrile framework. This chemotype is also structurally homologous to the first reported class of dual sulfatase/aromatase inhibitors, which are based on the 4-[benzyl(1,2,4 triazol-4-yl)amino]benzonitrile framework [24,25].

Before proceeding to chemical synthesis and biological evaluation of anticancer activity, our strategy involved generating a broad set of virtual analogues and assessing their molecular properties through computational modeling. This approach enables the identification and prioritization of the most promising candidates, ensuring that only the compounds predicted to exhibit the highest inhibitory potential are advanced to laboratory synthesis and subsequent biological assays.

## 2. Results

A total of 216 docking entries (99 designed scaffolds enumerated as syn/anti configurational pairs, plus nine additional carbamate analogs, also in syn/anti forms) were evaluated against STS.

**Figure 1:**
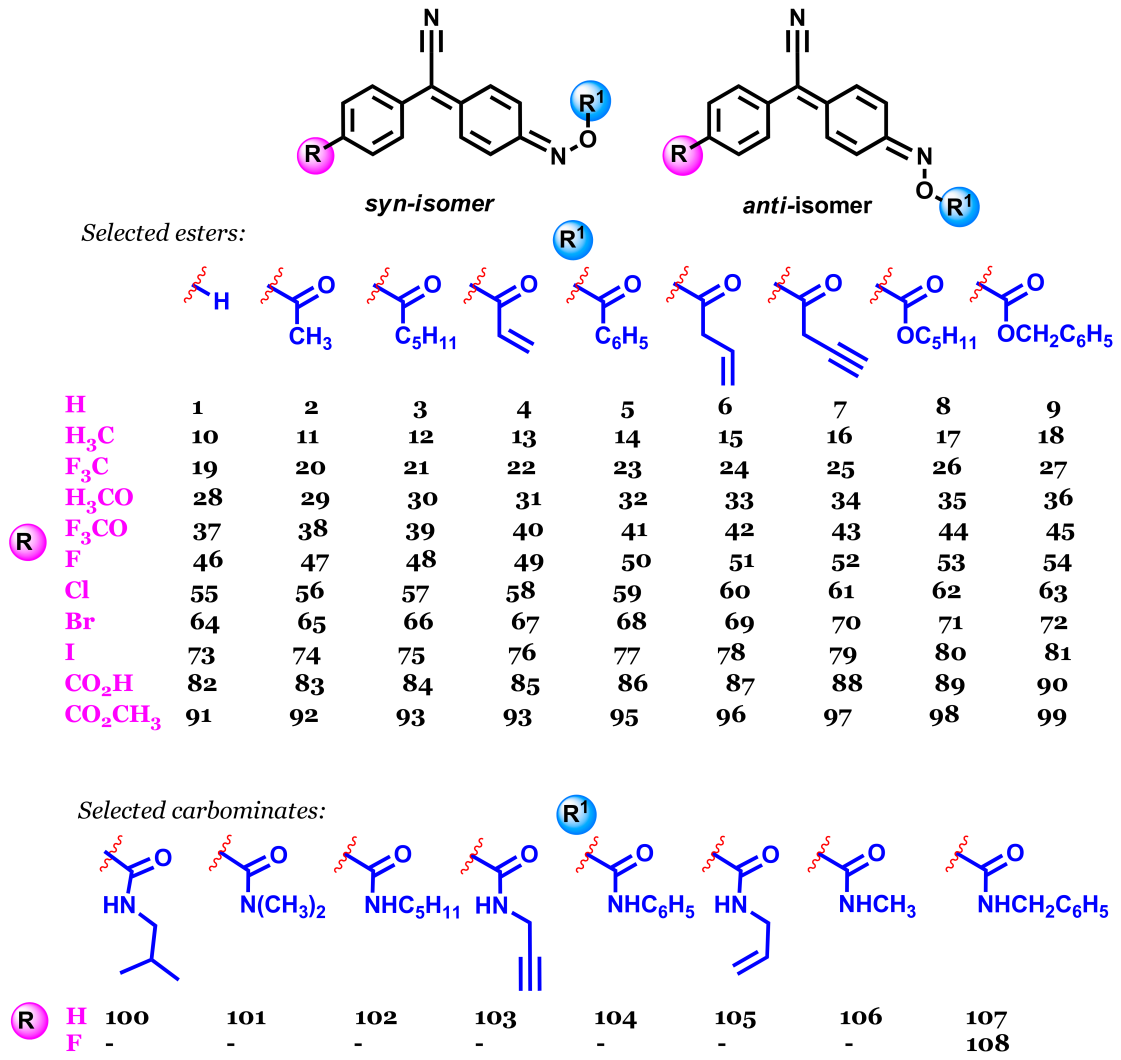
Virtual library of compounds based on the scaffold 2-(4-hydroxyiminocyclohexa-2,5-dien-1-ylidene)-2-phenylacetonitrile as potential sulfatase modulators.

Figure 1 Across the entire set, Vina binding scores for STS ranged from −9.726 to −7.077 (a more negative value means stronger predicted affinity). Paired comparison per molecule indicated that the anti-configuration was better (more negative STS score) in 55.6% of cases, with a mean (anti−syn) difference of −0.038. Thus, the two configurations are broadly comparable, with only a marginal advantage to the anti-isomer overall (Table 1).

These SBRs are consistent with the structural features of the hydrophobic pocket surrounding the FGly–Ca^2+^ catalytic region of STS, which is known to accept bulky hydrophobes that mimic steroidic motifs; our purely in silico data here align with that expectation without implying covalency for this scaffold. All AutoDock Vina 1.2.7 scores for the entire series are provided in the Supplementary Material.

Among all screened analogues, compound 45 (syn; R^1^ = OCF_3_, R^2^ = CO_2_CH_2_Ph) delivered the most favorable docking score against human steroid sulfatase (STS), with a Vina binding energy of −9.726 kcal·mol^−1^. In the validated docking model of STS (PDB 8EG3; active-site formylglycine (FGly) and Ca^2+^ retained), the ligand adopts an extended conformation that spans from the catalytic pocket into the neighboring hydrophobic channel.

**Figure 2:**
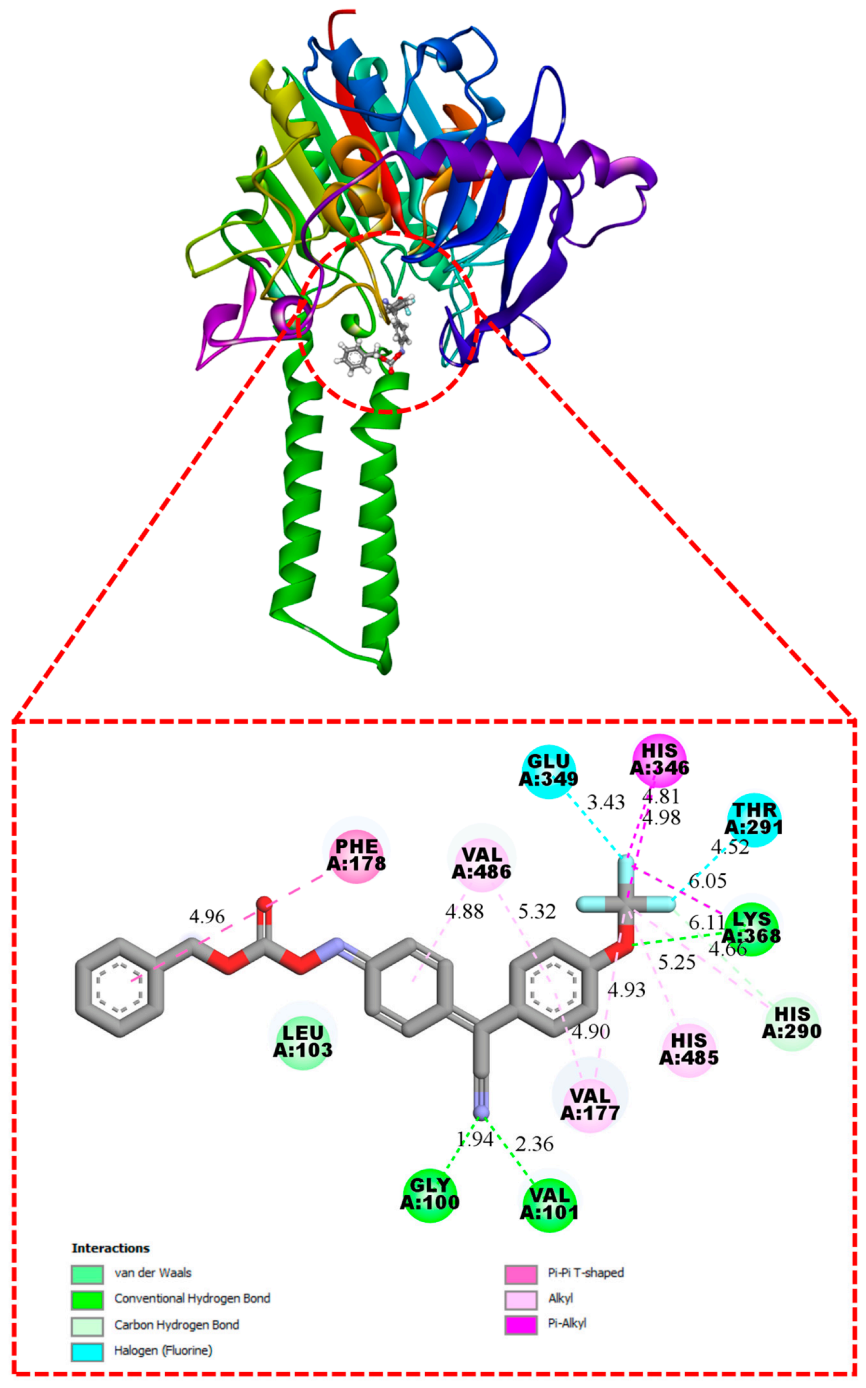
Binding pose and 2D interaction scheme of compound 45 (syn form) in the active site of STS (PDB 8EG3).

**Figure 3:**
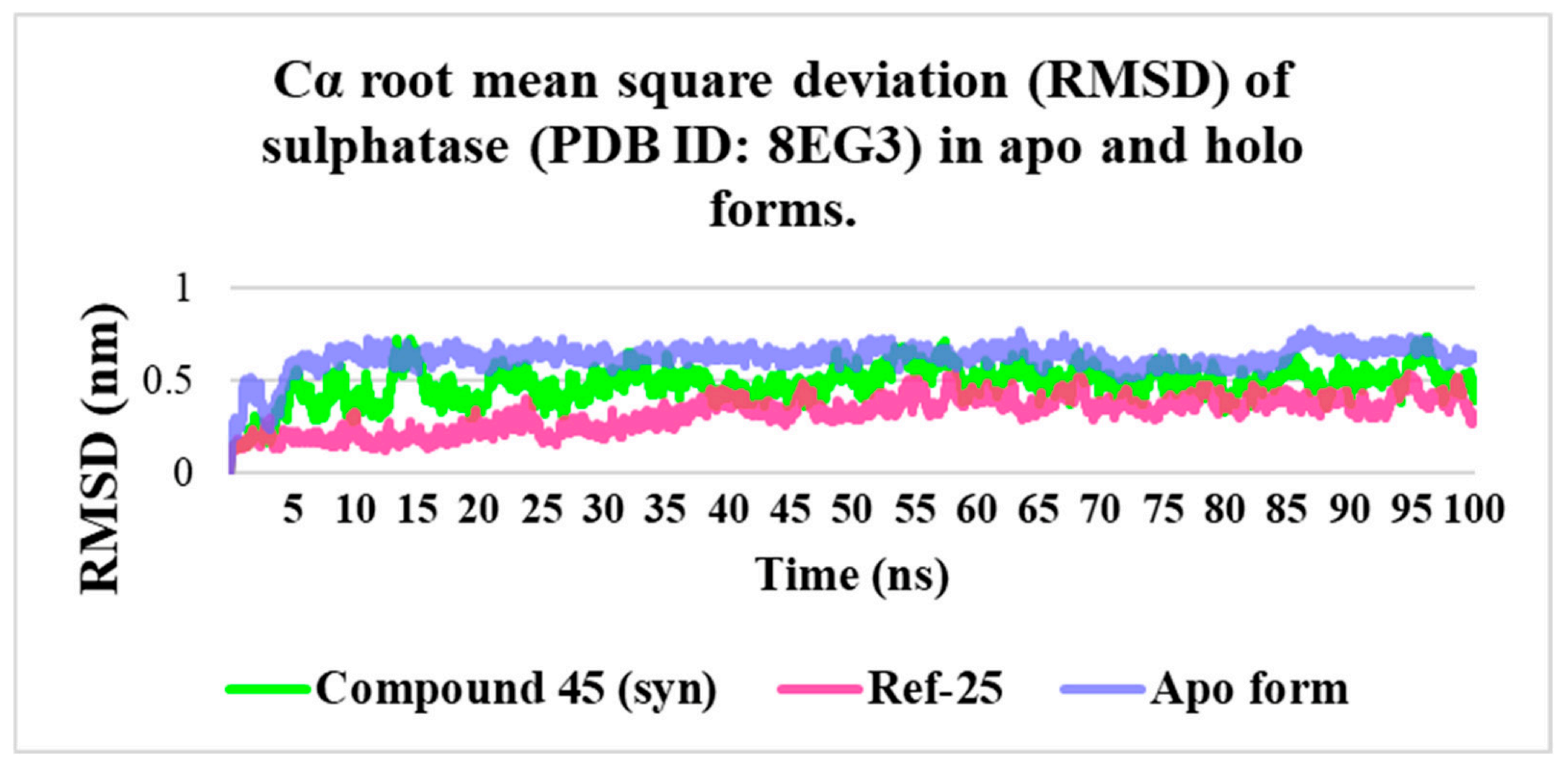
Cα RMSD (protein backbone) root mean square deviation (RMSD) profiles of steroid sulfatase (STS; PDB ID: 8EG3) in the apo form and in complexes with compound 45 (syn) and Ref-25 during molecular dynamics simulation.

The oxime–nitrile/phenoxy headgroup of 45 orients toward the Ca^2+^ proximal cluster (HIS290, HIS346, LYS368, GLU349), establishing a small network of conventional hydrogen bonds and carbon hydrogen bonds with HIS346 and GLU349 (inter heavy atom distances in the ~3.4–3.6 Å range in the interaction map), and additional polar contacts to HIS290/LYS368/THR291 that help anchor the pose at the mouth of the catalytic cavity (Figure: 3D/2D maps). The OCF_3_ headgroup contributes a weak halogen contact (F···X) toward the Ca^2+^ adjacent region (annotated near LYS368/THR291 in the 2D diagram), further consolidating the orientation of the ligand’s polar end. These features are consistent with the known polar environment of the STS active site surrounding the FGly gem diol and Ca^2+^. The benzyl ester tail (R^2^ = CO_2_CH_2_Ph) projects into a lipophilic sub-pocket where it engages multiple π–alkyl/alkyl contacts with VAL101, LEU103, VAL177, VAL486 and a π–π T-shaped interaction with PHE178. The central aryl core further reinforces these contacts via π–alkyl interactions with HIS485/VAL177. Collectively, this hydrophobic/π stacking envelope rationalizes the strong preference for aryl bearing R^2^ substituents observed across the library and explains why benzyl derived tails rank among the best performers in STS docking (Figure 2).

The binding mode of 45 is consistent with the SBR trends observed across the series: electron withdrawing/polarizable R^1^ groups (e.g., OCF_3_, CF_3_) and aryl bearing R^2^ motifs (benzyl ester/benzoyl/phenyl carbamates) are favored, as they simultaneously (a) anchor the ligand near the FGly–Ca^2+^ region through modest polar/halogen contacts and (b) maximize hydrophobic and π stacking occupancy within the adjacent channel. The resulting complementarity provides a structural rationale for the top-ranked STS score of compound 45 and, more generally, for the high potency of OCF_3_/aryl tail combinations in this scaffold.

Top-ranking compounds are 45-syn (CF_3_O/CO_2_CH_2_Ph), 86-anti (CO_2_H/COPh), 41-syn (CF_3_O/COPh), 23-syn (CF_3_/COPh). These combine very strong STS scores with chemically diverse R^1^/R^2^ patterns. Carbamate/urea analogs with phenyl appendages (R^2^ = OCONHCH_2_C_6_H_5_ or OCONHC_6_H_5_):108-syn/anti, 107-anti, 104-syn (STS −9.26 to −9.48).

### 2.1. Molecular Dynamics

Molecular dynamics (MD) simulations were carried out for steroid sulfatase (STS) in three states: the apo enzyme and the holo complexes with compound 45 (syn) and the reference ligand Ref-25, to assess the impact of ligand binding on the conformational dynamics of STS. Trajectories were analyzed using protein backbone RMSD, ligand RMSD (pose stability), residue-wise RMSF, hydrogen bond persistence, and the radius of gyration (Rg), providing a complementary view of global stability, local flexibility, compactness, and intermolecular contact retention.

Protein backbone RMSD was computed using Cα atoms (after least-squares fitting to the initial structure) to monitor global conformational stability. The protein backbone RMSD profiles indicate that ligand binding reduces large-scale conformational deviations relative to the apo form. The apo STS trajectory exhibits the highest RMSD values over the simulation, consistent with increased backbone mobility. In contrast, both ligand-bound systems display lower RMSD values and reach stable plateaus after the initial equilibration period, supporting an overall stabilizing effect of ligand binding. Among the holo systems, the Ref-25 complex shows the lowest RMSD values, suggesting the most pronounced reduction in global protein fluctuations under the simulated conditions. The STS–compound 45 (syn) complex also demonstrates improved stability compared with the apo form, although with slightly higher RMSD values than the reference complex. Overall, these results suggest that both ligands stabilize STS relative to the apo enzyme, with Ref-25 conferring the strongest global stabilization in this simulation (Figure 3). 

**Figure 4:**
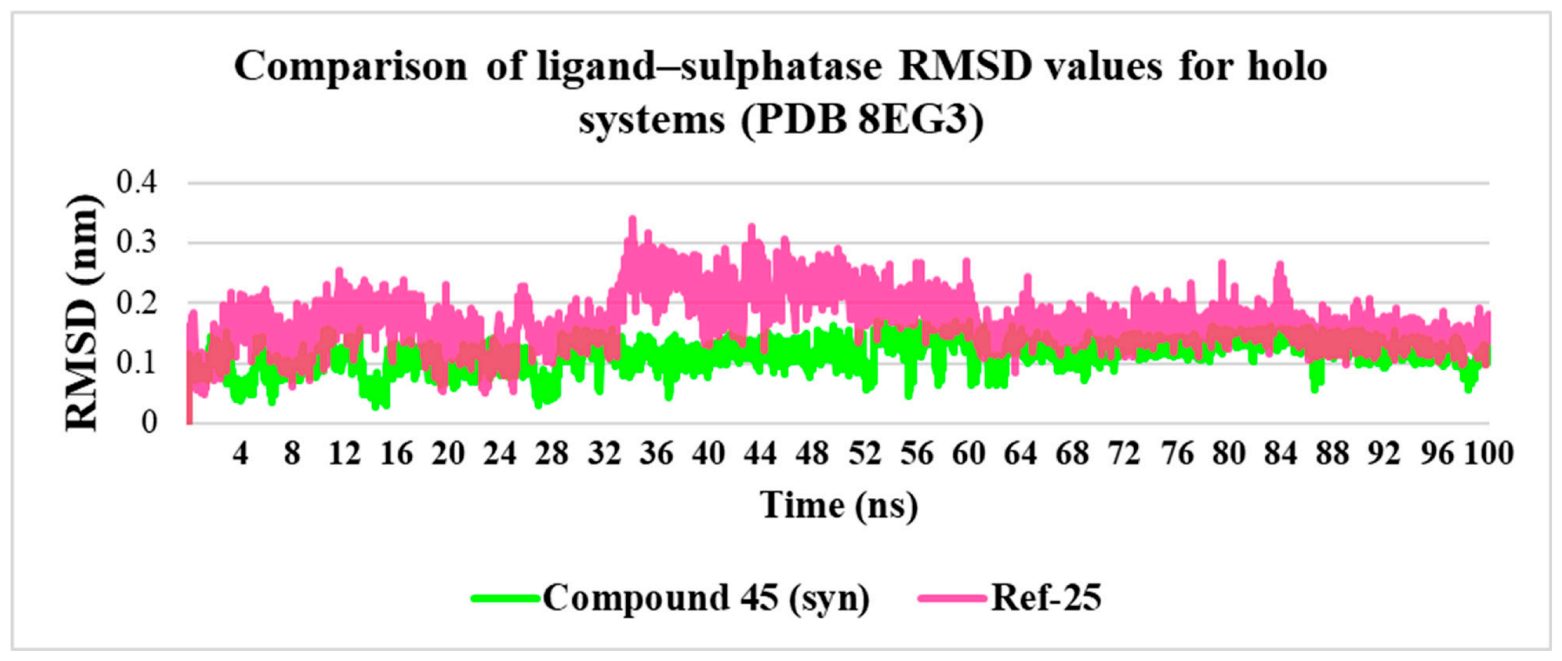
Ligand RMSD profiles for STS holo complexes with compound 45 (syn) and the reference ligand Ref-25 over 100 ns of MD simulation (PDB ID: 8EG3).

The RMSD analysis of ligand movement within the binding site indicates stable binding poses for both holo complexes throughout the simulation (Figure 3). Ligand RMSD values remain below ~0.3 nm for most of the trajectory, with no evidence of sustained large-scale displacement, suggesting that both ligands retain their binding-site positioning. Notably, compound 45 (syn) exhibits a consistently lower ligand RMSD profile than Ref-25, indicating reduced positional variability and a more stable bound conformation under the simulated conditions. In contrast, Ref-25 shows moderately higher RMSD values and a transient increase around ~35 ns, which is consistent with a short-lived conformational readjustment within the binding pocket rather than ligand dissociation (Figure 4).

**Figure 5:**
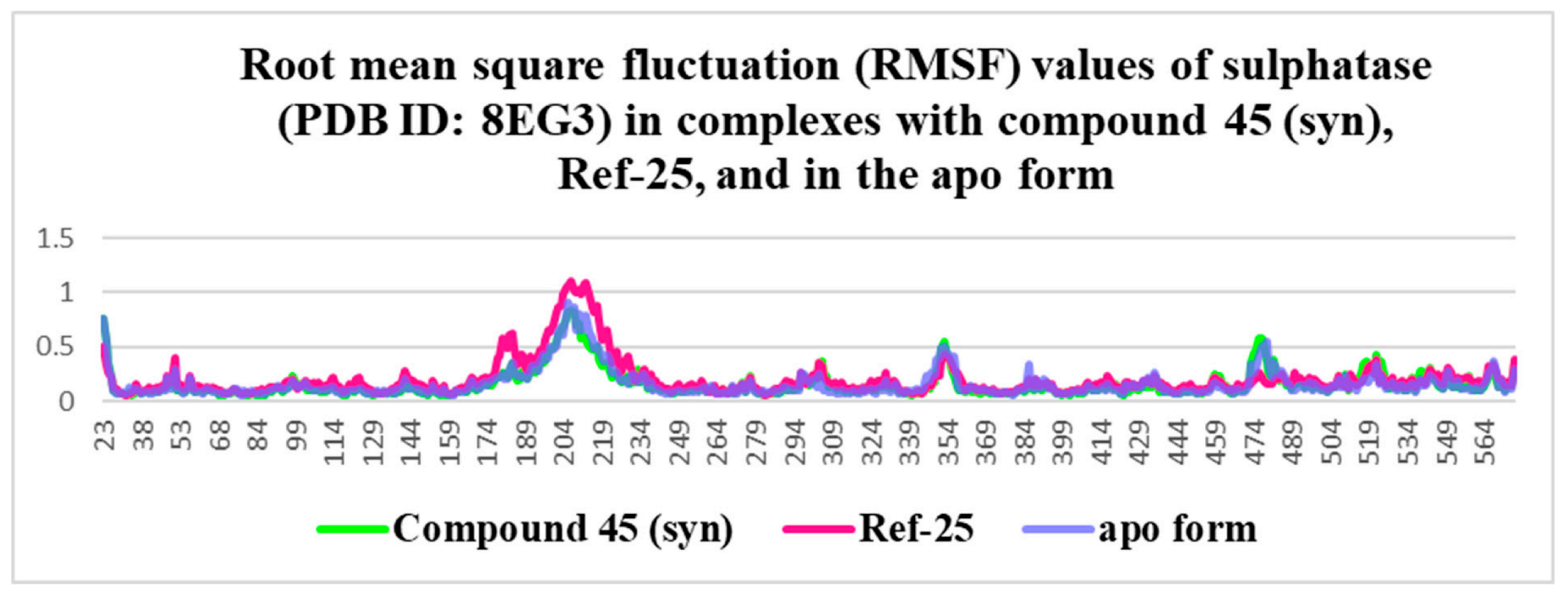
Root mean square fluctuation (RMSF) profiles of steroid sulfatase (STS; PDB ID: 8EG3) in the apo form and in complexes with compound 45 (syn) and Ref-25, showing residue-wise flexibility during molecular dynamics simulation.

The RMSF profiles provide insight into residue-level flexibility of STS in the apo and ligand-bound states (Figure 5). Overall, the three systems display broadly comparable fluctuation patterns along the sequence, indicating that ligand binding does not uniformly rigidify the enzyme. The most pronounced difference is observed in the region around ~200–230, where the Ref-25-bound complex exhibits the highest RMSF peak, whereas the STS–45 (syn) complex shows lower or comparable fluctuations relative to the apo form. Outside this region, only modest and localized differences are apparent, with both holo trajectories largely tracking the apo baseline.

Taken together, the RMSF analysis suggests that ligand binding modulates local dynamics in a site- and segment-dependent manner rather than producing a global reduction of flexibility. The slightly attenuated fluctuations observed for STS–45 (syn) in the major peak region are consistent with stable binding-site engagement inferred from docking, where compound 45 is predicted to form polar contacts (HIS346, GLU349, HIS290, LYS368, THR291) and hydrophobic/π-type interactions (VAL101, LEU103, VAL177, VAL486, PHE178, HIS485), which may contribute to maintaining a more constrained local environment.

**Figure 6:**
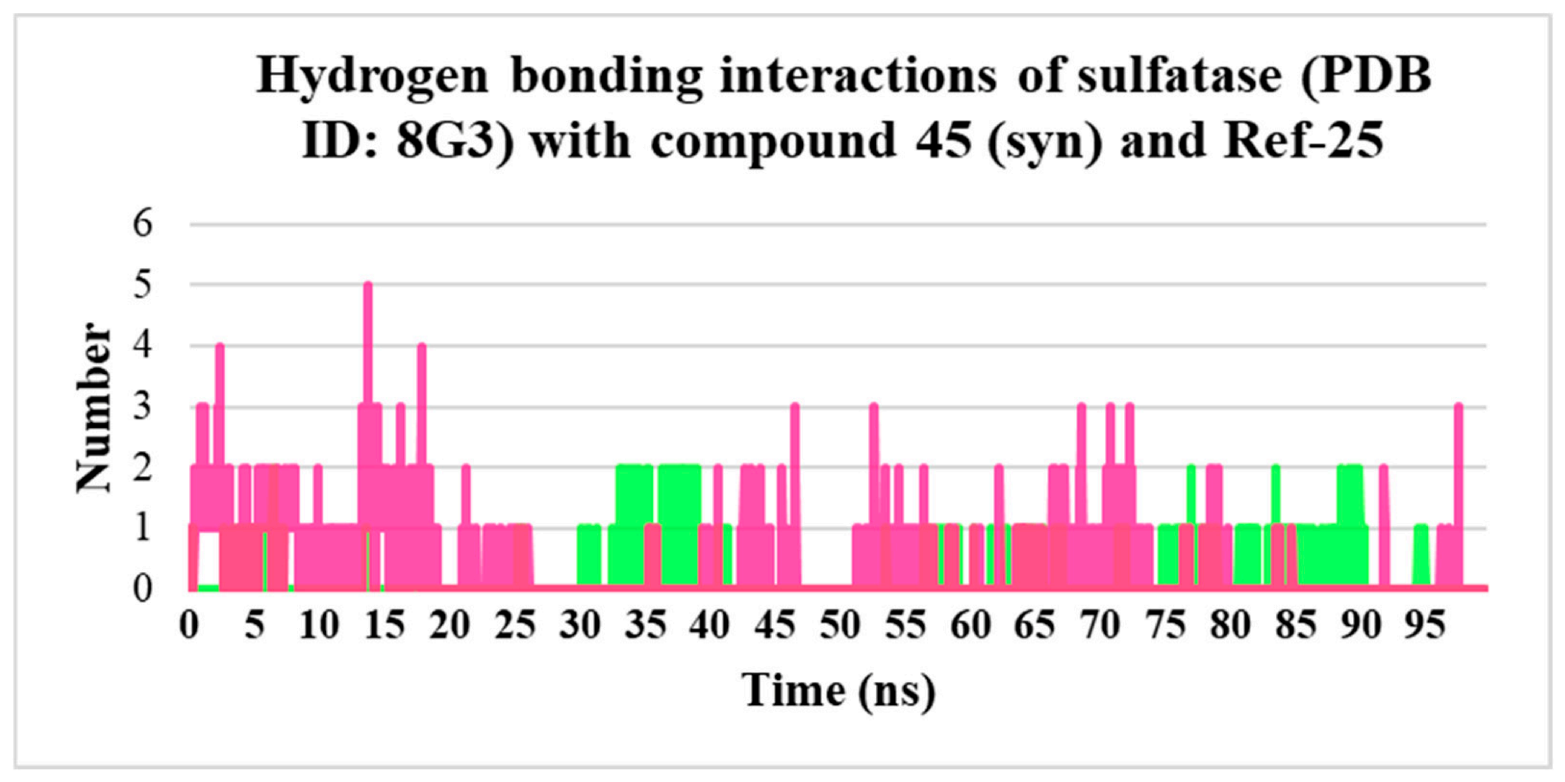
Hydrogen Bond Interactions of Sulfatase with Compound 45 (syn)—green-colored vs. Ref-25—pink-colored.

Hydrogen bond analysis further supports the stability of the ligand-bound states by characterizing the persistence and temporal pattern of polar contacts during the simulation. As shown in Figure 5, both ligands form hydrogen bonds with STS throughout the trajectory, although with distinct time-dependent profiles. Ref-25 exhibits a higher number of hydrogen bonds in the early stage of the simulation (approximately the first 20–25 ns), including several short-lived peaks, whereas its hydrogen-bonding becomes more intermittent thereafter. In contrast, compound 45 (syn) shows fewer high-amplitude peaks but displays a more consistent presence of low-count hydrogen bonds (typically 1–2) across the later portion of the simulation, indicating sustained polar engagement without evidence of ligand displacement (Figure 6).

This behaviour is consistent with the docking-derived interaction pattern of compound 45 (syn), which suggested a small network of polar contacts in the catalytic-site environment, potentially contributing to maintaining a stable binding pose over time.

Hydrogen bond occupancy analysis showed that Ref-25 formed a more persistent H-bond network The most stable contacts for Ref-25 was observed for THR180, ARG184, ARG98, PHE178, HIS485, VAL177, and GLY181, whereas 45 (syn) demonstrated lower overall occupancy, with contacts mostly observed for VAL101, GLY181, VAL177, GLY100, THR99, TRP550, HIS290, PHE178, and HIS485. Thus, the stability of 45-syn in the STS binding site seems to rely less on long-lived hydrogen bonds and more on transient polar contacts together with hydrophobic/π interactions in the adjacent binding pocket (Table 2).

Analysis of the radius of gyration (Rg) provides insight into the overall compactness of STS in the apo and ligand-bound states (Figure 6). The apo form consistently exhibits the lowest Rg values throughout the simulation, indicating a more compact baseline conformation. In contrast, both ligand-bound systems display slightly higher Rg values, suggesting a modest expansion of the protein structure upon ligand binding.

Among the holo systems, the STS–compound 45 (syn) complex maintains intermediate Rg values, whereas the Ref-25-bound complex generally exhibits the highest Rg and more pronounced fluctuations, particularly in the mid-simulation region (~30–45 ns). Despite these differences, all systems remain relatively stable over time, with no evidence of progressive unfolding or large-scale structural destabilization.

**Figure 7:**
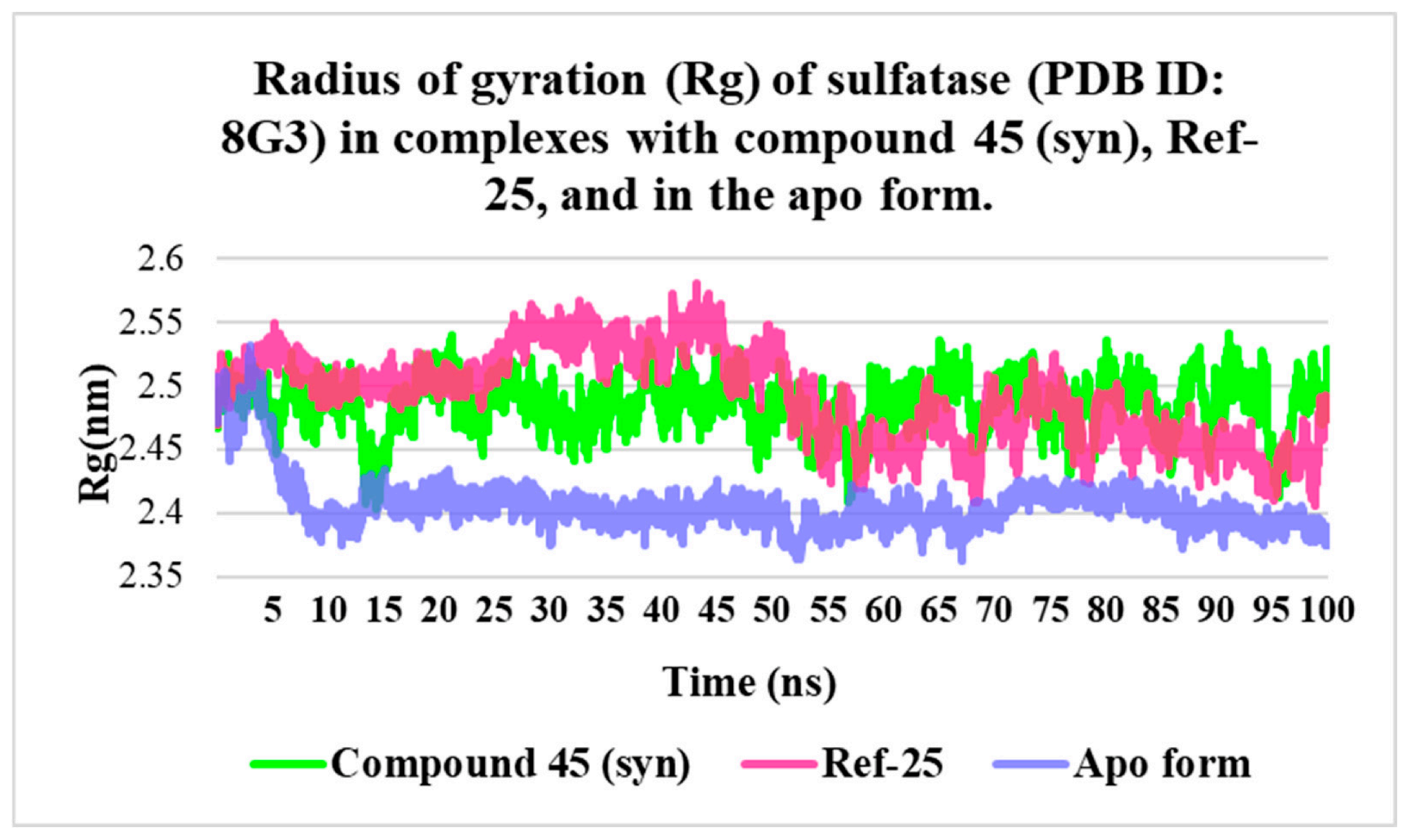
Radius of gyration (Rg) of steroid sulfatase (STS; PDB ID: 8EG3) in the apo form and in complexes with compound 45 (syn) and Ref-25 over a 100 ns MD simulation. In summary, MD simulations suggest that both ligands tend to stabilize STS compared to the apo form of the enzyme. Ref-25 gives the strongest stabilization and shows more persistent hydrogen bonding overall. Compound 45-syn, on the other hand, stays stably bound even though its H-bond occupancy is lower. Its stability seems to rely more on a mix of transient polar contacts and hydrophobic/π-type interactions inside the binding pocket, rather than on one or two very strong, long-lived hydrogen bonds.

Overall, the Rg profiles indicate that ligand binding induces subtle conformational adjustments that slightly reduce compactness while preserving global structural integrity. The intermediate behaviour of compound 45 (syn) is consistent with its balanced interaction pattern observed in docking and MD analyses, suggesting stable binding without extensive perturbation of the protein fold (Figure 7).

### 2.2. MM-PBSA Calculation

To further evaluate the thermodynamic stability of the complexes, the binding free energies (ΔGbind) were estimated using the end-point Molecular Mechanics Poisson–Boltzmann Surface Area (MM-PBSA) approach. The analysis was performed on representative snapshots extracted from the equilibrated MD trajectories of STS in complex with compound 45 (syn) and the reference ligand Ref-25. In this framework, (ΔGbind) is calculated as the sum of molecular mechanics terms (van der Waals and electrostatics) and implicit-solvent contributions, divided into polar (Poisson–Boltzmann) and non-polar (surface area) components.

The calculated ΔG values indicate favorable binding for both ligands, with ΔG = −13.41 ± 2.92 kcal/mol for the STS–compound 45 (syn) complex and a slightly more negative ΔG = −15.80 ± 3.10 kcal/mol for STS–Ref-25. For compound 45 (syn), the energetic profile is dominated by a strong van der Waals term (−23.37 ± 2.03 kcal/mol), complemented by electrostatic interactions (−5.41 ±2.04 kcal/mol). These findings are consistent with the hydrophobic pi-contacts and polar anchoring observed during docking and MD simulations.

The favorable gas-phase interactions are partially counterbalanced by a polar solvation penalty (+15.37 ± 3.31 kcal/mol), while the non-polar solvation term (−1.84 ± 0.04 kcal/mol) provides modest additional stabilization. Consequently, the net solvation contribution for 45(syn) is +15.36 ± 3.31 kcal/mol, highlighting that desolvation costs offset a portion of the interaction energy and emphasizing the critical role of hydrophobic packing in maintaining binding affinity.

A similar energetic partitioning was observed for Ref-25, characterized by stronger van der Waals (−25.70 ± 2.10 kcal/mol) and electrostatic (−7.00 ± 2.10 kcal/mol) contributions. These are accompanied by a larger polar solvation penalty (+18.60 ± 3.40 kcal/mol) and a non-polar term of +2.00 ± 0.08 kcal/mol, resulting in an overall solvation energy of +16.60 ± 3.40 kcal/mol. The slightly more favorable ΔGbind for Ref-25 correlates with its more pronounced stabilizing effect on the STS structure, as evidenced by lower backbone RMSD values relative to both the apo form and the 45(syn) complexes.

Overall, the MM-PBSA results provide an energetics-based validation of the docking and MD rankings. Both ligands form stable complexes driven primarily by dispersive contacts, while polar solvation acts as the main energetic barrier to binding. It should be noted that these end-point estimates are interpreted comparatively within the same protocol to prioritize candidates rather than as absolute experimental affinities. All energetic components and total *ΔG* values are summarized in Table 3.

## 3. Predicted ADMET Profiles of the Tested Set

The in silico ADMET profiling of the top-ranked steroid sulfatase inhibitors was performed to evaluate their pharmacokinetic behavior and potential toxicity risks at an early stage of drug development. The analyzed compounds generally fall within an acceptable molecular weight range (326–452 g/mol), consistent with drug-like chemical space. Most derivatives exhibit moderate to high lipophilicity (cLogP ≈ 3.3–5.0), which is favorable for membrane permeability, although the highest values may suggest a tendency toward reduced aqueous solubility. The topological polar surface area (TPSA) values (62–100 Å^2^) indicate a balance between permeability and polarity, supporting oral bioavailability for the majority of compounds. In agreement with this, most candidates are predicted to have high gastrointestinal absorption, with only a few exceptions (e.g., compounds 45 and 27), which may require structural optimization.

None of the investigated molecules are predicted to be P-glycoprotein substrates, suggesting a lower risk of efflux-mediated drug resistance. Blood–brain barrier (BBB) permeability is generally absent, which may be advantageous for minimizing central nervous system-related side effects in anticancer therapy. Importantly, all compounds comply with Lipinski’s rule of five, showing zero violations, thus supporting their drug-likeness. The hydrogen bond donor/acceptor profiles further indicate favorable intermolecular interaction potential without excessive polarity.

Toxicological predictions based on ProTox-III suggest moderate safety profiles, with most compounds classified within toxicity classes 3–5. The predicted LD50 values span from 275 to 5000 mg/kg, indicating variability in acute toxicity but generally acceptable margins for further optimization. Notably, compound 41 demonstrates the highest predicted safety (LD50 = 5000 mg/kg, class 5), while several compounds fall into class 3, suggesting the need for cautious interpretation. Overall, the ADMET analysis supports the selection of these compounds as promising candidates for further development, while also highlighting specific derivatives that may benefit from structural refinement to improve pharmacokinetic and safety properties (Table 4).

The predicted lead compound 45 (syn) demonstrated low gastrointestinal (GI) absorption, which may represent a limitation for potential oral administration. However, if these predictions are confirmed experimentally, further structural optimization may be required to improve its ADMET profile.

Since the ADMET parameters were estimated in silico, these results should be considered preliminary. Experimental pharmacokinetic and toxicological studies will be necessary to validate the predicted properties and assess the compound’s suitability for further development.

## 4. Materials and Methods

### 4.1. Molecular Docking

In order to identify anti-sulfatase activities, in silico simulations were performed, including molecular docking and molecular dynamics studies. A virtual library consisting of 99 derivatives, plus nine additional carbamate analog compounds, was constructed. We created the focused combinatorial library by systematically varying two regions, R^1^ and R^2^. At R^1^, we introduced groups with diverse electronic profiles: H, CH_3_, OCH_3_, F, Cl, Br, I, CF_3_, OCF_3_, CO_2_H, and CO_2_Me. This set was chosen to cover electron-donating and electron-withdrawing effects, halogen-bonding potential, and polar functionality.

The R^2^ position was modified with ester, ketone, carbamate, and urea-type fragments, including both aliphatic and aromatic fragments. We focused on terminal phenyl-containing groups because the STS crystal structure shows a hydrophobic channel near the catalytic FGly–Ca^2+^ region that can accommodate bulky lipophilic substituents. Aromatic fragments were therefore expected to strengthen hydrophobic and π-stacking interactions in the pocket.

Since the oxime group can exist as syn- and anti-geometrical isomers, we generated both configurations and used them in the screening. This decision ensured more complete coverage of conformational space and allowed us to assess the effect of oxime geometry on binding inside STS by in silico approaches.

All forms were docked to the STS structure (PDB ID: 8EG3). The catalytically active residue Cys75 was represented in its post-translationally modified formylglycine (FGly) state, for subsequent docking and molecular dynamics simulations. AutoDock Vina 1.2.7 was employed for the in silico docking studies due to its high computational efficiency, accuracy, and established reliability for docking to sulfatase enzymes [26]. The docking grid was centered at coordinates x = 69.795, y = −11.330, and z = 35.178, with dimensions of 22.5 × 22.5 × 22.5 Å, ensuring full coverage of the target binding site. The exhaustiveness parameter was set to 32 to increase the accuracy of the conformational search. A maximum of 10 binding poses was generated for each ligand, and the energy range was set to 4 kcal/mol. The resulting docking poses were ranked according to their predicted binding affinities, and the highest-scoring conformations were selected for further analysis. During the system preparation, all polar hydrogens were added to the amino acid residues, and Kollman charges were assigned and distributed across all atoms. As part of the validation procedure, redocking of the native ligands was performed. Due to the absence of a crystallized native inhibitor, we performed redocking of N-acetylglucosamine (NAG), which is present in the structure. The obtained RMSD values, all below 2 Å, indicate that the selected parameters are capable of accurately reproducing ligand positions within the constructed models [27,28]. Considering the need for a suitable benchmark to evaluate compounds from our proposed set, and to ensure the use of a compound that does not act via the formation of covalent irreversible bonds, we selected N-[3-hydroxy-2-nitroestra-1,3,5(10)-trien-17-yl]-3-(trifluoromethyl)benzene-1-sulfonamide (Ref-25) as a reference ligand. This compound has been experimentally confirmed as an inhibitor of steroid sulfatase while not forming covalent bonds [29].

### 4.2. Molecular Dynamics Simulations and MM/PBSA Analysis

Molecular dynamics (MD) simulations were performed to investigate the structural stability and dynamic behavior of steroid sulfatase (STS) in different states. The simulations were performed using the SIBIOLed server with the GROMACS 2021 molecular dynamics engine [30]. Three systems were considered: the apo form of STS, the complex with the top-ranked compound identified from docking studies, and the complex with a reference ligand. 

All simulations were conducted employing AMBER-based force fields supported by the server, including ff14SB for the protein and the general AMBER force field (GAFF) for ligand parameterization [31]. Each system was simulated under physiological conditions at 303 K in the presence of 0.15 M NaCl to mimic ionic strength. The total simulation time was 100 ns, and trajectory data were recorded as 5000 frames to ensure sufficient sampling.

Both NVT and NPT equilibration steps were performed using the leap-frog md integrator with a timestep of 2 fs. Each equilibration step consisted of 250,000 steps, corresponding to 500 ps. During NVT equilibration, the temperature was maintained at 300 K using the V-rescale thermostat with a coupling time constant of 0.1 ps, while pressure coupling was not applied. During NPT equilibration, the same V-rescale thermostat was used, and pressure was maintained at 1 bar using isotropic Berendsen pressure coupling with a coupling time constant of 2 ps and a compressibility of 4.5 × 10^−5^ bar^−1^.

Long-range electrostatic interactions were treated using the particle mesh Ewald (PME) method with a real-space Coulomb cutoff of 1.2 nm, Fourier spacing of 0.16 nm, and PME interpolation order of 4. Van der Waals interactions were treated using a cut-off scheme with a force-switch modifier, with switching starting at 1.0 nm and a cutoff of 1.2 nm. The Verlet cutoff scheme and periodic boundary conditions in all directions were applied. Bond constraints were handled using the LINCS algorithm with lincs-order 4 and lincs-iter 1, while SETTLE was used for water molecules.

Trajectory analyses included evaluation of root mean square deviation (RMSD), root mean square fluctuation (RMSF), hydrogen bond formation, and radius of gyration (Rg), enabling comparative assessment of structural stability, flexibility, and compactness.

Binding free energy calculations were performed using the Molecular Mechanics Poisson–Boltzmann Surface Area (MM/PBSA) method implemented within the simulation workflow.

Binding energy values were calculated from 51 frames extracted at 2 ns intervals from the 100 ns production trajectories. The reported binding energies include van der Waals, electrostatic, polar solvation, and non-polar solvation contributions. Entropic contributions were not included in these calculations; therefore, the reported ΔG values should be interpreted as approximate relative binding energies rather than rigorous absolute thermodynamic free energies [32].

### 4.3. In Silico ADMET Prediction

The pharmacokinetic and toxicity profiles of the selected compounds were evaluated using in silico ADMET prediction tools. Physicochemical properties, lipophilicity (cLogP), topological polar surface area (TPSA), gastrointestinal absorption, blood–brain barrier (BBB) permeability, P-glycoprotein (P-gp) substrate recognition, and drug-likeness parameters were calculated using the SwissADME online platform. Drug-likeness was assessed according to Lipinski’s rule of five, including molecular weight, hydrogen bond donors and acceptors, and lipophilicity criteria [28].

Toxicological properties, including median lethal dose (LD50) and toxicity class, were predicted using the ProTox-III web server (version 3.0). The toxicity classification was based on the Globally Harmonized System (GHS), categorizing compounds into classes according to their predicted acute oral toxicity [33].

All compounds were submitted as SMILES strings, and default parameters were applied in both platforms. The combined use of these tools enabled a comprehensive early-stage evaluation of pharmacokinetic behavior and safety profiles, supporting the prioritization of compounds with favorable ADMET characteristics for further study.

## 5. Conclusions

The series of 2-(4-hydroxyiminocyclohexa-2,5-dien-1-ylidene)-2-phenylacetonitrile derivatives was evaluated as potential non-covalent inhibitors of steroid sulfatase, using modern in silico tools. A virtual library of 216 *syn*/*anti-* isomers was screened against the human STS crystal structure using molecular docking. The calculated binding affinities ranged from −7.077 to −9.726 kcal·mol^−1^, indicating generally favorable interactions with the STS active site. Among the tested compounds, 45-syn showed the best docking score and was the only candidate whose affinity approached the values observed for the reference ligands. Docking analysis revealed that its binding was supported by polar contacts near the catalytic FGly–Ca^2+^ region, together with hydrophobic and π-type interactions within the adjacent binding pocket. The SBR analysis indicated that electron-withdrawing substituents at R^1^ and aromatic or benzyl-containing groups at R^2^ are beneficial for improved STS binding. Molecular dynamics simulations confirmed that ligand binding stabilizes the STS structure compared with the apo form. The 45-syn–STS complex maintained stable interactions during simulation and showed reduced local flexibility in functionally relevant regions of the enzyme.

MM/PBSA calculations further supported the favorable binding profile of the selected ligand–STS complexes. In silico ADMET prediction suggested that the most promising candidates possess acceptable drug-like properties and no major predicted pharmacokinetic liabilities. Overall, compound 45-syn represents the most promising hit identified in this study.

These results at first indicate potential significant inhibitory activity of quinone methide oximes towards steroid sulfatase, and provide a rational basis for the further synthesis, experimental validation, and optimization of this scaffold as a potential new class of STS inhibitors for hormone-dependent breast cancer therapy.

## Figures and Tables

**Table 1:** Top steroid sulfatase docking hits. Ranking is based on the best-scoring isomer per compound against human steroid sulfatase (STS; PDB 8EG3). Scores are AutoDock Vina 1.2.7 binding energies in kcal·mol.

Rank	Compound ID	Isomer	R^1^	R^2^	AutoDock Vina Score (kcal·mol^−1^)
**1**	**45**	**syn**	**CF_3_O**	**CO_2_CH_2_Ph**	**−9.726**
2	86	anti	CO_2_H	COPh	−9.666
3	108	syn	F	OCONHCH_2_Ph	−9.482
4	41	syn	CF_3_O	COPh	−9.436
5	23	syn	CF_3_	COPh	−9.428
6	27	anti	CF_3_	CO_2_CH_2_Ph	−9.342
7	107	anti	H	OCONHCH_2_Ph	−9.336
8	104	syn	H	OCONHPh	−9.259
9	5	syn	H	COPh	−9.252
10	36	syn	CH_3_O	CO_2_CH_2_Ph	−9.218
11	32	syn	CH_3_O	COPh	−9.178
12	50	anti	F	COPh	−9.177
13	21	anti	CF_3_	COC_5_H_11_	−9.100
14	68	anti	Br	COPh	−9.068
15	77	syn	I	COPh	−9.038
16	25	anti	CF_3_	CO_2_CH_2_CCH	−8.979
17	18	anti	CH_3_	CO_2_CH_2_Ph	−8.958
18	14	syn	CH_3_	COPh	−8.947
19	84	anti	CO_2_H	COC_5_H_11_	−8.933
20	95	syn	CO_2_Me	COPh	−8.929
	**Ref-25**				**−10.658**

R^1^. The following mean STS scores (ascending = stronger) were observed: CF_3_ (−8.547) > CO_2_H (−8.441) > CH_3_O (−8.326) ≈ CF_3_O (−8.322) > H (−8.122) > CO_2_Me (−8.098) > F (−8.048) > CH_3_ (−8.036) > Br (−7.951) ≈ I (−7.951) > Cl (−7.904). The data favor strongly electron-withdrawing and/or polarizable R^1^ groups (CF_3_, CF_3_O, CO_2_H) over halogens heavier than F. R^2^. Mean STS scores grouped by R^2^ indicate aryl-bearing carbamates/ureas and benzoyl derivatives as optimal: OCONHCH_2_C_6_H_5_ (−9.364) > OCONHC_6_H_5_ (−9.116) > COPh (−9.099) > CO_2_CH_2_Ph (−8.729), followed by aliphatic esters/carbamates (−7.60 to −8.16 range). Thus, introducing a terminal phenyl ring at R^2^—either as benzoyl, benzyl ester, or phenyl carbamate—consistently enhances STS binding.

**Table 2:** Hydrogen bond occupancy of the main ligand–STS contacts during molecular dynamics simulations.

Residue	Ref-25 Occupancy (%)	45-syn Occupancy (%)
THR180	14.55	1.24
VAL101	0.02	7.76
ARG184	6.36	0.00
GLY181	1.64	4.28
ARG98	4.00	0.06
VAL177	2.02	3.38
ILE226	3.16	0.00
PHE178	3.00	1.50
HIS485	2.86	1.30
GLY100	0.04	2.14
THR99	0.30	1.76
TRP550	0.40	1.58
HIS290	0.00	1.38
PHE488	0.08	1.12
VAL486	0.02	0.92
THR291	0.00	0.24
LYS368	0.00	0.10
HIS346	0.00	0.02

**Table 3:** MM-PBSA Binding Free Energy and Energy Decomposition for STS in Complex with Compound 45(syn) and Ref-25.

Complex	ΔG	Van der Waal Energy	Electrostatic Energy	Polar Solvation Energy	Non-Polar Solvation Energy	Solvation Energy
STS-com 45 (syn)	−13.41 ± 2.92	−23.37 ± 2.03	−5.41 ± 2.04	15.37 ± 3.31	−1.84 ± 0.04	15.36 ± 3.31
STS–Ref-25	−15.80 ± 3.10	−25.70 ± 2.10	−7.00 ± 2.10	18.60 ± 3.40	2.00 ± 0.08	16.60 ± 3.40

**Table 4:** In silico ADMET properties of selected STS inhibitors predicted using SwissADME and ProTox-III.

Ranking	Cmpd	MW	cLogP	TPSA	HBD/HBA	GI Abs	BBB	P-gp	Lipinski	LD_50_	Tox Cl.
**1**	**45**	440.37	4.77	80.91	0/9	Low	No	No	0	1000	4
2	86	370.36	3.36	99.75	1/6	High	No	No	0	275	3
3	108	356.37	3.89	71.68	0/5	High	Yes	No	0	275	3
4	41	410.35	4.65	71.68	0/8	High	No	No	0	5000	5
5	23	394.35	4.82	62.45	0/7	High	No	No	0	275	3
6	27	424.37	4.94	71.68	0/9	Low	No	No	0	275	3
7	107	336.38	4.00	71.68	0/5	High	No	No	0	275	3
8	104	326.35	3.77	62.45	0/4	High	Yes	No	0	275	3
9	5	326.35	3.77	62.45	0/4	High	Yes	No	0	275	3
10	36	386.40	3.88	80.91	0/6	High	No	No	0	2300	5
11	32	356.37	3.77	71.68	0/5	High	Yes	No	0	1190	4
12	50	344.34	4.08	62.45	0/5	High	Yes	No	0	275	3
13	21	388.38	5.04	62.45	0/7	High	Yes	No	0	934	4
14	68	405.24	4.39	62.45	0/4	High	Yes	No	0	275	3
15	77	452.24	4.42	62.45	0/4	High	Yes	No	0	275	3
16	25	356.30	3.97	62.45	0/7	High	Yes	No	0	275	3
17	18	370.40	4.23	71.68	0/5	High	No	No	0	275	3
18	14	340.37	4.12	62.45	0/4	High	Yes	No	0	275	3
19	84	364.39	3.58	99.75	1/6	High	No	No	0	275	3
20	95	384.38	3.76	88.75	0/6	High	No	No	0	275	3

Abbreviations: Cmpd, compound; MW, molecular weight (g/mol); cLogP, calculated octanol/water partition coefficient; TPSA, topological polar surface area (Å^2^); HBD/HBA, hydrogen bond donors/acceptors; GI Abs, gastrointestinal absorption; BBB, blood–brain barrier permeability.

## Data Availability

All structural coordinates used in this study were retrieved from the RCSB Protein Data Bank (PDB; https://www.rcsb.org) accessed on 1 April 2026; the corresponding accession codes are listed in the Materials and Methods section. This study did not generate new experimental data. Simulation input files, processed trajectories, and other relevant datasets supporting the findings can be requested from the corresponding authors under reasonable conditions.

## Supplementary Materials

The following supporting information can be downloaded at: https://www.mdpi.com/article/10.3390/molecules31152612/s1. Table S1: Predicted AutoDock Vina 1.2.7 binding energies (kcal/mol) of the virtual library compounds toward steroid sulfatase (STS) calculated for syn and anti oxime conformers.

## Abbreviations

The following abbreviations are used in this manuscript:

ADMET	Absorption, Distribution, Metabolism, Excretion, and Toxicity
AKT	Protein Kinase B (PKB)
BBB	Blood–Brain Barrier
DHEAS	Dehydroepiandrosterone Sulfate
E1	Estrone
E2	Estradiol
E1S	Estrone Sulfate
ER	Estrogen Receptor
ER+	Estrogen Receptor-positive
ERα	Estrogen Receptor alpha
ESR1	Estrogen Receptor 1 (gene)
FGly	Formylglycine
GHS	Globally Harmonized System
HER2	Human Epidermal Growth Factor Receptor 2
IGF1R	Insulin-like Growth Factor 1 Receptor
MAPK	Mitogen-Activated Protein Kinase
MD	Molecular Dynamics
MM/PBSA	Molecular Mechanics Poisson–Boltzmann Surface Area
NAG	N-acetylglucosamine
PDB	Protein Data Bank
PI3K	Phosphoinositide 3-kinase
Rg	Radius of Gyration
RMSD	Root-Mean-Square Deviation
RMSF	Root-Mean-Square Fluctuation
SBR	Structure–Binding Relationship(s)
SMILES	Simplified Molecular-Input Line-Entry System
STS	Steroid Sulfatase
TPSA	Topological Polar Surface Area
