# Supplementary material for: In Silico Design and Evaluation of Quinone Methide Oxime Derivatives as Potential Non-Covalent Steroid Sulfatase Inhibitors
Source: Molecules. 2026 Jul 27;31(15):2612. doi: 10.3390/molecules31152612 (PMC13468570; doi:10.3390/molecules31152612)
Supplement: Supplementary file 1 [file molecules-31-02612-s001.zip › molecules-4418988-supplementary.pdf]

Supplementary Materials

In Silico Design and Evaluation of Quinone Methide Oxime Derivatives as Potential Non-Covalent Steroid Sulfatase Inhibitors

Dmytro Khylyuk [1,*], Oleg M. Demchuk [2,*], Sergii Holota [3,4], Dagmara Otto-Ślusarczyk [5], Marta Struga [5], Franciszek Burdan [6] and Monika Wujec [1]

[1] Chair and Department of Organic Chemistry, Medical University of Lublin, ul. Chodźki 4a, 20-093 Lublin, Poland
[2] Faculty of Medicine, The John Paul II Catholic University of Lublin, Konstantynow 1J, 20-708 Lublin, Poland
[3] Department of Pharmaceutical, Organic and Bioorganic Chemistry, Danylo Halytsky Lviv National Medical University, 69 Pekarska St., 79010 Lviv, Ukraine; golota_serg@yahoo.com
[4] Molecular Design Center, Danylo Halytsky Lviv National Medical University, Pekarska 69, 79010 Lviv, Ukraine
[5] Department of Biochemistry, Medical University of Warsaw, Banacha 1, 02-097 Warsaw, Poland; dagmara.otto@wum.edu.pl (D.O.-Ś.)
[6] Department of Human Anatomy, Medical University of Lublin, 4 Jaczewski Street, 20-090 Lublin, Poland
* Correspondence: dmytro.khylyuk@umlub.edu.pl (D.K.); oleh.demchuk@kul.pl (O.M.D.)

Table of Contents

- List of predicted binding energies for the target virtual library................................S2-S6

Compound	R	R^1	Syn form binding energy (kcal/mol)	Anty form binding energy (kcal/mol)

number				
1	H	H	-7.547	-7.528
2	H	$COCH_3$	-7.732	-7.077
3	H	COC_5H_{11}	-8.177	-8.245
4	H	$COCHCH_2$	-7.569	-7.677
5	H	COPh	-9.252	-9.171
6	H	$COOCH_2CH=CH_2$	-7.701	-7.865
7	H	$COOCH_2C\equiv CH$	-7.901	-7.946
8	H	$COOC_5H_{11}$	-7.342	-7.565
9	H	$COOCH_2Ph$	-8.190	-8.641
10	H_3C	H	-7.841	-7.349
11	H_3C	$COCH_3$	-8.094	-7.309
12	H_3C	COC_5H_{11}	-7.670	-7.979
13	H_3C	$COCHCH_2$	-7.772	-7.988
14	H_3C	COPh	-8.947	-8.933
15	H_3C	$COOCH_2CH=CH_2$	-7.516	-7.870
16	H_3C	$COOCH_2C\equiv CH$	-7.534	-8.338
17	H_3C	$COOC_5H_{11}$	-8.015	-8.065
18	H_3C	$COOCH_2Ph$	-8.464	-8.958
19	F_3C	H	-8.347	-7.893
20	F_3C	$COCH_3$	-8.503	-7.772
21	F_3C	COC_5H_{11}	-8.187	-9.100
22	F_3C	$COCHCH_2$	-8.279	-8.770
23	F_3C	COPh	-9.428	-9.393

24	F_3C	$COOCH_2CH{=}CH_2$	-8.137	-8.901
25	F_3C	$COOCH_2C{\equiv}CH$	-8.178	-8.979
26	F_3C	$COOC_5H_{11}$	-7.960	-8.264
27	F_3C	$COOCH_2Ph$	-8.409	-9.342
28	H_3CO	H	-7.774	-8.378
29	H_3CO	$COCH_3$	-8.173	-7.961
30	H_3CO	COC_5H_{11}	-7.576	-8.492
31	H_3CO	$COCHCH_2$	-8.103	-8.420
32	H_3CO	COPh	-9.178	-9.149
33	H_3CO	$COOCH_2CH{=}CH_2$	-7.724	-8.461
34	H_3CO	$COOCH_2C{\equiv}CH$	-7.744	-8.206
35	H_3CO	$COOC_5H_{11}$	-8.069	-8.090
36	H_3CO	$COOCH_2Ph$	-9.218	-9.155
37	F_3CO	H	-8.377	-7.748
38	F_3CO	$COCH_3$	-8.802	-7.548
39	F_3CO	COC_5H_{11}	-8.095	-7.676
40	F_3CO	$COCHCH_2$	-8.760	-7.724
41	F_3CO	COPh	-9.436	-8.914
42	F_3CO	$COOCH_2CH{=}CH_2$	-8.325	-7.699
43	F_3CO	$COOCH_2C{\equiv}CH$	-8.331	-7.560
44	F_3CO	$COOC_5H_{11}$	-8.629	-7.711
45	F_3CO	$COOCH_2Ph$	-9.726	-8.728
46	F	H	-7.815	-7.691
47	F	$COCH_3$	-7.992	-7.516

48	F	COC_5H_{11}	-7.716	-8.256
49	F	$COCHCH_2$	-7.762	-7.963
50	F	COPh	-9.017	-9.177
51	F	$COOCH_2CH{=}CH_2$	-7.581	-7.738
52	F	$COOCH_2C{\equiv}CH$	-7.871	-7.950
53	F	$COOC_5H_{11}$	-7.901	-7.805
54	F	$COOCH_2Ph$	-8.356	-8.764
55	Cl	H	-7.829	-7.243
56	Cl	$COCH_3$	-7.984	-7.276
57	Cl	COC_5H_{11}	-7.485	-7.861
58	Cl	$COCHCH_2$	-7.721	-7.882
59	Cl	COPh	-8.872	-8.737
60	Cl	$COOCH_2CH{=}CH_2$	-7.437	-7.725
61	Cl	$COOCH_2C{\equiv}CH$	-7.510	-8.066
62	Cl	$COOC_5H_{11}$	-7.574	-7.787
63	Cl	$COOCH_2Ph$	-8.432	-8.855
64	Br	H	-7.892	-7.203
65	Br	$COCH_3$	-8.005	-7.333
66	Br	COC_5H_{11}	-7.794	-7.977
67	Br	$COCHCH_2$	-7.721	-7.896
68	Br	COPh	-8.947	-9.068
69	Br	$COOCH_2CH{=}CH_2$	-7.504	-7.771
70	Br	$COOCH_2C{\equiv}CH$	-7.534	-7.935
71	Br	$COOC_5H_{11}$	-7.451	-7.842

72	Br	$COOCH_2Ph$	-8.395	-8.858
73	I	H	-7.959	-7.242
74	I	$COCH_3$	-8.092	-7.279
75	I	COC_5H_{11}	-7.825	-7.805
76	I	$COCHCH_2$	-7.481	-7.881
77	I	COPh	-9.038	-8.956
78	I	$COOCH_2CH{=}CH_2$	-7.492	-7.836
79	I	$COOCH_2C{\equiv}CH$	-7.551	-7.937
80	I	$COOC_5H_{11}$	-7.435	-7.933
81	I	$COOCH_2Ph$	-8.474	-8.903
82	COOH	H	-7.999	-8.359
83	COOH	$COCH_3$	-8.164	-8.141
84	COOH	COC_5H_{11}	-8.143	-8.933
85	COOH	$COCHCH_2$	-8.140	-8.439
86	COOH	COPh	-9.136	-9.666
87	COOH	$COOCH_2CH{=}CH_2$	-8.169	-8.627
88	COOH	$COOCH_2C{\equiv}CH$	-8.111	-8.743
89	COOH	$COOC_5H_{11}$	-8.256	-7.849
90	COOH	$COOCH_2Ph$	-8.430	-8.629
91	$COOCH_3$	H	-7.964	-7.509
92	$COOCH_3$	$COCH_3$	-8.185	-7.629
93	$COOCH_3$	COC_5H_{11}	-7.761	-7.916
94	$COOCH_3$	$COCHCH_2$	-8.158	-7.972
95	$COOCH_3$	COPh	-8.929	-8.845

96	$COOCH_3$	$COOCH_2CH{=}CH_2$	-7.873	-8.017
97	$COOCH_3$	$COOCH_2C{\equiv}CH$	-7.947	-7.929
98	$COOCH_3$	$COOC_5H_{11}$	-8.113	-7.897
99	$COOCH_3$	$COOCH_2Ph$	-8.240	-8.873
100	H	$CONHCH_2CH(CH_3)_2$	-8.16	-7.985
101	H	$CON(CH_3)_2$	-7.907	-7.712
102	H	$CONHC_5H_{11}$	-7.436	-7.828
103	H	$CONCH_2C{\equiv}CH$	-7.828	-7.794
104	H	CONHPh	-9.259	-8.972
105	H	$CONCH_2CH{=}CH_2$	-7.944	-7.767
106	H	$CONHCH_3$	-7.605	-7.598
107	H	$CONHCH_2Ph$	-9.221	-9.336
108	F	$CONHCH_2Ph$	-9.482	-9.415
